# Association of the Clinical and Radiographic Findings at Onset With Future Joint Destruction in Patients With Rheumatoid Arthritis

**DOI:** 10.7759/cureus.39428

**Published:** 2023-05-24

**Authors:** Kenji Takami, Shigeyoshi Tsuji, Masataka Nishikawa, Hajime Owaki

**Affiliations:** 1 Orthopaedic Surgery, Nippon Life Hospital, Osaka, JPN; 2 Rheumatology, Japan Community Healthcare Organization Osaka Hospital, Osaka, JPN; 3 Orthopaedic Surgery, Japan Community Healthcare Organization Osaka Hospital, Osaka, JPN

**Keywords:** joint tenderness, joint swelling, rheumatoid arthritis, x-ray analysis, squeeze test, joint destruction

## Abstract

Objectives: Since inflammation can cause joint destruction in patients with rheumatoid arthritis (RA), it is assumed that joints that are symptomatic at onset are at higher risk of joint destruction; however, this theory remains controversial. This study aimed to investigate whether the progression of joint destruction in hands and feet could be predicted from the clinical and radiographic findings at onset.

Methods: This study included 75 patients who visited our hospital within one year after the onset of RA with at least 12 months of follow-up. We examined the positive predictive value (PPV) and the sensitivity of the clinical findings (swelling, tenderness, and squeeze test) and joint destruction at onset for the progression of joint destruction.

Results: Sixty joints (45 metacarpophalangeal and proximal interphalangeal joints, 15 metatarsophalangeal joints) exhibited progressive structural destruction during the study course. Both the PPV and the sensitivity of the clinical findings for the progression of joint destruction were low; however, only the sensitivity of the squeeze test for the feet was high. The PPV of joint destruction at onset was higher than the clinical findings, and the sensitivity of joint destruction at onset was as high as the squeeze test for the feet.

Conclusions: Regular follow-up with imaging is necessary regardless of symptoms and joint destruction at the onset. Adding the squeeze test for feet to routine clinical practice may help predict the risk of joint destruction for the feet.

## Introduction

The treatment goals for patients with rheumatoid arthritis (RA) include functional, structural, and clinical remission. Clinical remission is a prerequisite for achieving structural remission, whereas structural and clinical remission are critical for achieving functional remission [1]. Structural remission refers to the suppression of joint destruction, which associate with many factors, such as clinical symptoms, blood test data, and composite scores [2-9]. Since inflammation can cause joint destruction and relapses reportedly often occur in the same joint, it is assumed that joints that are symptomatic at onset are at higher risk for destruction later; however, this remains controversial [10]. This study aimed to investigate whether the progression of joint destruction could be predicted using the clinical and radiographic findings in the hands and feet at onset.

## Materials and methods

Patients characteristics and evaluation

Of the patients who visited the Japan Community Healthcare Organization Osaka Hospital, Osaka, Japan, within one year after RA onset, 75 (2,250 joints) who completed at least 12 months of follow-up were included (mean follow-up duration, 67.5 months).

We examined the swelling and tenderness of the metacarpophalangeal (MCP), proximal interphalangeal (PIP), and metatarsophalangeal (MTP) joints as well as the squeeze test results and joint destruction during the study course. The squeeze test is a useful technique for the clinical evaluation of a group of small, adjacent joints such as the MCP and MTP joints as described previously [11,12]. For the squeeze test, a strongly positive result was defined as quickly retracting a hand or foot, a positive result was defined as feeling pain, and a weakly positive result was defined as feeling discomfort in this study. The progression of joint destruction was defined as increasing the grade of Larsen classification. We examined the positive predictive value (PPV) and the sensitivity of the clinical findings (swelling, tenderness, and squeeze test) and joint destruction at onset for the progression of joint destruction.

The Ethics Review Committee of Japan Community Healthcare Organization Osaka Hospital approved the study (approval number: 2023-001 (4/19/2023).

Statistics

The chi-square test was used to analyze all values among the groups. Statistical analyses were performed using GraphPad Prism 9 (GraphPad Software Inc., San Diego, California, United States).

## Results

Demographic characteristics and radiographic changes

The patients' basic characteristics are described in Table 1. Sixty joints (45 MCP and PIP, 15 MTP) displayed progressive structural destruction during the study course. Table 2 shows the joint destruction at onset and at the last follow-up, which shows a worsening in number and Larsen grade. The association of the swelling, tenderness, and the results of the squeeze test with joint destruction at onset was low (Table 3); however, in the feet, the association of the squeeze test with joint destruction was relatively high (62.5%).

**Table 1:** Basic characteristics of the patients Data are shown as mean ± standard deviation, unless otherwise specified. BMI: body mass index; RF: rheumatoid factor; ACPA: anti-cyclic citrullinated peptide antibody

Number of Cases	75
Age (years)	58.0±14.6
Female (%)	65.3
Height (cm)	159.2±9.4
Body weight (kg)	57.3±11.0
BMI	22.5±3.1
RF positivity (%)	81.1
ACPA positivity (%)	74.6
Duration of disease (months)	6.1±2.9
Duration of follow-up (months)	67.5±26.5

**Table 2:** Radiographic changes from the onset to the last follow-up Data are shown as the number, unless otherwise specified.

Onset	Hands	Feet
The number of patients with joint destruction	28	16
The number of joint destruction	68	25
Larsen grade		
Grade Ⅰ	21	9
Grade Ⅱ	43	11
Grade Ⅲ	4	3
Grade Ⅳ	0	2
Last follow-up		
The number of patients with joint destruction	32	17
The number of joint destruction	82	32
Larsen grade		
Grade Ⅰ	0	0
Grade Ⅱ	62	20
Grade Ⅲ	12	5
Grade Ⅳ	8	7

**Table 3:** Association of joint destruction and experiment at onset Data are shown as n (%), unless otherwise specified. S+: joint swelling positive; S-: joint swelling negative; T+: joint tenderness positive; T-: joint tenderness negative; Sq±: squeeze test weak positive

Larsen grade	1 or more		Zero	
	Hands	Feet	Hands	Feet
S+T+	13/68 (19.1)	0/25 (0)	101/1432 (7.1)	12/725 (1.7)
S+T-	10/68 (14.7)	0/25 (0)	58/1432 (4.1)	2/725 (0.3)
S-T+	3/68 (4.4)	3/25 (12.0)	92/1432 (6.4)	99/725 (13.7)
Sq± or more	10/25 (40.0)	15/24 (62.5)	54/125 (43.2)	52/126 (41.3)

PPV of clinical findings at onset

The PPV for the progression of joint destruction was 8.8% for the hands and 0% for the feet when both joint swelling and tenderness were present at onset (Table 4). Similarly, the PPV for swelling alone and tenderness alone were 11.8% and 4.2% for the hands and 0% and 4.9% for the feet, respectively (Table 4). In the squeeze test, the PPV for the progression of joint destruction was 28.6% for the hands and 21.4% for the feet in strongly positive cases and 24.2% for the hands and 14.6% for the feet in positive cases (Table 4).

**Table 4:** Positive predictive value of each experiment for progression of joint destruction Data are shown as n (%), unless otherwise specified; The positive predictive values of all the tests were compared with a chi-square test for both hands and feet. S+: joint swelling positive; S-: joint swelling negative; T+: joint tenderness positive; T-: joint tenderness negative; Sq++: squeeze test strongly positive; Sq+: squeeze test positive

	S+T+	S+T-	S-T+	Sq++	Sq+	p-value
Hands	10/114（8.8）	8/68（11.8）	4/95（4.2）	4/14（28.6）	8/33（24.2）	0.0030
Feet	0/12（0）	0/2（0）	5/102（4.9）	3/14（21.4）	6/41（14.6）	0.0830

Sensitivity of clinical findings at onset

The sensitivity, that is the percentage of symptoms at onset in joints with progressive joint destruction, was 22.2% for the hands and 0% for the feet in both joint swelling and tenderness (Table 5). The sensitivities of swelling alone and tenderness alone were 17.8% and 8.9% for the hands and 0% and 33.3% for the feet, respectively (Table 5). The sensitivity for swelling or tenderness was 48.9% for the hands and 33.3% for the feet (Table 5). In the hands, the PPV and the sensitivity for swelling tended to be predominant for predicting the progression of joint destruction compared to tenderness. Conversely, in the feet tenderness was predominant, although all the percentages were low. The overall sensitivity of the squeeze test at onset, including weakly positive, positive, and strongly positive, was 61.9% for the hands and 84.6% for the feet (Table 5).

**Table 5:** Sensitivity of each experiment for progression of joint destruction Data are shown as n (%), unless otherwise specified; The sensitivities of all the tests were compared with a chi-square test for both hands and feet. S+: joint swelling positive; S-: joint swelling negative; T+: joint tenderness positive; T-: joint tenderness negative; Sq++: squeeze test strongly positive; Sq+: squeeze test positive; Sq±: squeeze test weak positive

	S+T+	S+T-	S-T+	S+ or T+	Sq++	Sq+	Sq± or more	p-value
Hands	10/45（22.2）	8/45（17.8）	4/45（8.9）	22/45（48.9）	4/21（19.0）	8/21（38.1）	13/21（61.9）	<0.0001
Feet	0/15（0）	0/15（0）	5/15（33.3）	5/15（33.3）	3/13（23.1）	6/13（46.2）	11/13（84.6）	0.0095

PPV and sensitivity of joint destruction at onset

When there was joint destruction at onset, the PPV was 41.2% for the hands and 44.0% for the feet (Table 6), each of which was higher than the PPV of the clinical findings. The PPV increased to 60.0% and 100% when combined with swelling in the hands and tenderness in the feet, respectively (Table 6). However, caution is needed in interpretation due to the small number of cases. The sensitivity was 62.2% for the hands, and 73.3% for the feet (Table 6), each of which was also higher than the sensitivity of the clinical findings.

**Table 6:** PPV and sensitivity of joint destruction and each experiment at onset for the progression of joint destruction Data are shown as n (%), unless otherwise specified. D+: joint destruction; S+: joint swelling positive; S-: joint swelling negative; T+: joint tenderness positive; T-: joint tenderness negative; Sq++: squeeze test strongly positive; Sq+: squeeze test positive; PPV: positive predictive value; NA: not available

	D+	D+S+T+	D+S+T-	D+S-T+
PPV				
Hands	28/68 (41.2)	6/13 (46.2)	6/10 (60.0)	1/3 (33.3)
Feet	11/25 (44.0)	NA	NA	3/3 (100)
Sensitivity				
Hands	28/45 (62.2)	NA	NA	NA
Feet	11/15 (73.3)	NA	NA	NA

## Discussion

Clinical symptoms at RA onset are not necessarily predictive of the progression of joint destruction. As above the association of joint symptoms with the progression of joint destruction is weak, which means we should pay attention not only to those joints with symptoms at onset; rather, all joints should be evaluated regularly by imaging tests. However, the foot squeeze test had a high sensitivity for the progression of joint destruction. Although the squeeze test is reportedly useful as a predictor of potential progression to RA and an indicator of RA disease activity, there are no reports on its usefulness as a predictor of joint destruction [12-17]. As revealed in this study, the foot squeeze test is highly sensitive with respect to subsequent MTP joint structural destruction and may be useful as a routine test in daily practice.

In this study, the structural destruction on the initial x-ray was found to be more informative than the clinical findings on the initial examination, with higher values for the PPV and the sensitivity for the progression of joint destruction. Characteristically, swelling in the hands and tenderness in the feet were found to be associated with the progression of joint destruction. The reason for the opposite results for the hands and feet may be that nonspecific tenderness tends to be in the hands leading to a false positive. In addition, it is difficult to detect swelling and rare to detect nonspecific tenderness in the feet, which may lead to the above result.

This study had limitations. First, the treatment initiation timings and the follow-up durations differed among patients. Second, the imaging evaluation was performed by a single investigator. Third, the location and timing of imaging were decided by the attending physician alone. Fourth, RA inflammation control was not considered.

## Conclusions

Regular follow-up with imaging tests is necessary for patients with RA regardless of symptoms and joint destruction at onset. Our findings suggest that adding the foot squeeze test to routine clinical practice may help predict the risk of joint destruction for the feet.
